# Impact of high-sensitivity cardiac troponin I assay imprecision on the safety of a single-sample rule-out approach for myocardial infarction

**DOI:** 10.1515/cclm-2024-1011

**Published:** 2024-09-20

**Authors:** Ziwen Li, Yong Yong Tew, Peter A. Kavsak, Kristin M. Aakre, Allan S. Jaffe, Fred S. Apple, Paul O. Collinson, Nicholas L. Mills, On behalf of the High-STEACS Trial Investigators

**Affiliations:** BHF Centre for Cardiovascular Science, University of Edinburgh, Edinburgh, UK; Department of Pathology and Molecular Medicine, McMaster University, Hamilton, ON, Canada; Department of Medical Biochemistry and Pharmacology and Department of Heart Disease, Haukeland University Hospital, Bergen, Norway; Department of Clinical Science, University of Bergen, Bergen, Norway; Departments of Cardiology and Laboratory Medicine and Pathology, Mayo Clinic, Rochester, MN, USA; Department of Laboratory Medicine and Pathology, Hennepin Healthcare/ HCMC, Minneapolis, MN, USA; Department of Laboratory Medicine and Pathology, University of Minnesota, Minneapolis, MN, USA; Cardiac Biomarkers Trials Laboratory, Hennepin Healthcare Research Institute, Minneapolis, MN, USA; St George’s University of London, London, UK; Usher Institute, University of Edinburgh, Edinburgh, UK

**Keywords:** imprecision, high-sensitivity cardiac troponin, myocardial infarction, single-sample rule-out

To the Editor,

High-sensitivity cardiac troponin (hs-cTn) assays facilitate identification of low-risk patients for immediate discharge and thus may help reduce emergency department (ED) overcrowding. The HiSTORIC trial evaluated implementation of a single-sample rule-out pathway using a hs-cTnI assay in 31,492 consecutive patients [1]. Following implementation, length of stay was reduced by 3 h and discharge from the ED increased by 21 % with no evidence of adverse events [1]. However, assay imprecision at the low hs-cTn thresholds deployed in single-sample rule-out pathways could impact performance [2]. There is presently little guidance on the allowable imprecision at lower concentration thresholds [3]. Here we model the impact of imprecision on misclassification and therefore the safety of a single-sample rule-out strategy for myocardial infarction (MI).

In 48,282 consecutive patients with suspected acute coronary syndrome (median age 61 [interquartile range 49–75] years, 47 % female) evaluated using a hs-cTnI assay (ARCHITECT_*STAT*_*,* Abbott Laboratories; Abbott Park, IL, USA) in the HighSTEACS trial [4], we simulated the effect of imprecision for a range of CVs at the rule-out threshold of <5 ng/L: 10 % (±0.5 ng/L), 20 % (±1 ng/L), 30 % (±1.5 ng/L), 40 % (±2 ng/L), 50 % (±2.5 ng/L), 60 % (±3 ng/L), 70 % (±3.5 ng/L), 80 % (±4 ng/L), 90 % (±4.5 ng/L), and 100 % (±5 ng/L). The *rnorm* function in R was used, setting the mean to the initial cTn concentrations at presentation (ground truth) and the standard deviation to the absolute change corresponding to each CV. The resulting concentrations were then rounded to the nearest whole numbers as used in clinical practice. To model the impact of imprecision, we repeated the simulation 100 times and estimated the proportion reclassified to higher risk (from <5 ng/L to ≥5 ng/L), to lower risk (from ≥5 ng/L to <5 ng/L), and overall, in patients with cTn concentrations below the 99th percentile at presentation. We subsequently calculated the likelihood of (i) a primary outcome of an adjudicated index diagnosis of type 1 MI, type 4b MI or type 4c MI and (ii) a secondary outcome of any myocardial injury on serial testing, in those potentially misclassified as low risk at presentation due to assay imprecision. The 95 % confidence intervals (95 % CI) were estimated using a binomial likelihood with an equal-tailed Jeffreys prior using the *binom. bayes* function in R.

**Figure 1:**
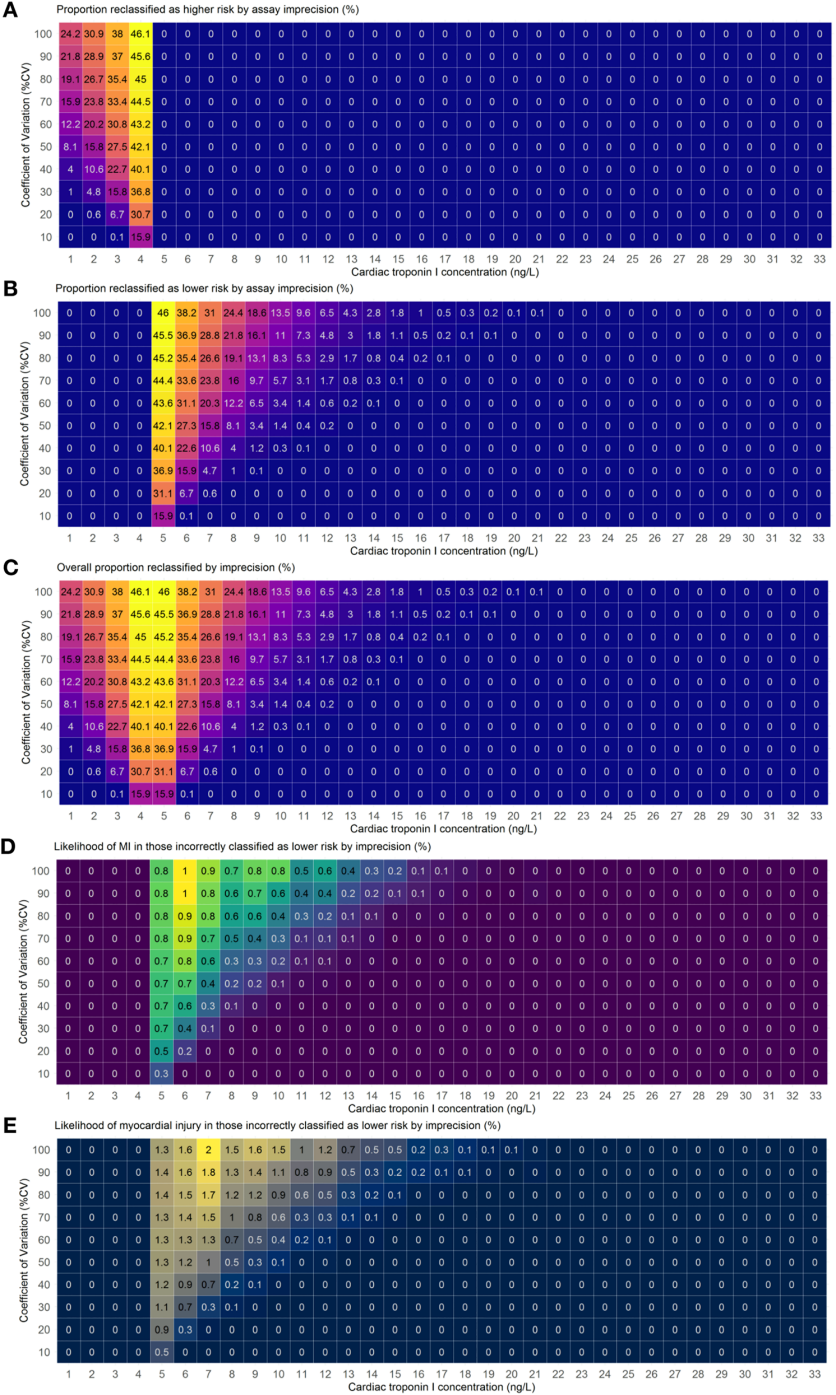
Estimated proportion of patients with suspected acute coronary syndrome reclassified due to hs-cTnI assay imprecision at the rule-out threshold of <5 ng/L and likelihood of MI or myocardial injury in those misclassified as a proportion of the total number of patients at each measured cardiac troponin concentration. (A) Proportion reclassified as higher risk by assay imprecision (%). (B) Proportion reclassified as lower risk by assay imprecision (%). (C) Overall proportion reclassified by imprecision (%). (D) Likelihood of MI in those incorrectly classified as lower risk by imprecision (%). (E) Likelihood of myocardial injury in those incorrectly classified as lower risk by imprecision (%). Patients with STEMI (n=925) were excluded from the modelling.

Based on our simulations, the effect of assay imprecision on the proportion reclassified from low to higher risk, which could result in unnecessary serial testing, increased as the CV and cTn concentrations increased from 1 to 4 ng/L (Figure 1). Similarly, the effect of assay imprecision on the proportion of reclassified from higher risk to low risk, which could result in harm, increased as the CV increased, and cTn concentrations decreased from 21 ng/L to 5 ng/L. Overall reclassification was depicted as a cone-shaped gradient. The likelihood of primary and secondary outcomes associated with reclassification from higher risk to low risk had a similar gradient.

For example, using an assay with a 10 % CV at the rule-out threshold of <5 ng/L, reclassification to low risk due to assay imprecision would occur in 15.9 % (95%CI 14.4%–17.5 %) and 0.1 % (95%CI 0%–0.4 %) patients with a measured cTn concentration of 5 ng/L and 6 ng/L, respectively (Table 1). The likelihood of having a MI in those reclassified due to imprecision who actually had a value of 5 ng/L when the measured concentration was<5 ng/L would be 0.3 % (95 %CI 0.1 %–0.5 %). Overall imprecision at this level would result in 0.01 % (95 %CI 0.01 %–0.03 %) of all patients evaluated being incorrectly classified as low risk who may have a missed index MI.

**Table 1:** Estimated proportion of patients with suspected acute coronary syndrome misclassified due to hs-cTnI assay imprecision (coefficient of variation (CV) from 10 to 50 %) at the rule-out threshold of <5 ng/L and the proportion of all patients evaluated (n=47,357) with missed MI or myocardial injury due to imprecision.

CV	Measured hs-cTnI concentration, ng/L	Proportion misclassified as low risk by imprecision % (95 %CI)	Likelihood of MI in those reclassified % (95 %CI)	Proportion of all patients with missed MI due to imprecision % (95 %CI)	Likelihood of myocardial injury in those reclassified % (95 %CI)	Proportion of all patients with missed myocardial injury due to imprecision % (95 %CI)
10 %	5	15.9 (14.4–17.5)	0.3 (0.1–0.5)	0.01 (0.01–0.03)	0.5 (0.2–0.8)	0.02 (0.01–0.04)
	6	0.1 (0–0.4)	0 (0–0.2)		0 (0–0.2)	
20 %	5	31.1 (29.2–33.1)	0.5 (0.3–0.9)	0.03 (0.02–0.05)	0.9 (0.6–1.4)	0.05 (0.03–0.07)
	6	6.7 (5.5–8)	0.2 (0–0.5)		0.3 (0.1–0.6)	
	7	0.6 (0.3–1.2)	0 (0–0.2)		0 (0–0.3)	
30 %	5	36.9 (34.9–39)	0.7 (0.4–1.1)	0.05 (0.03–0.07)	1.1 (0.8–1.7)	0.08 (0.06–0.11)
	6	15.9 (14.2–17.7)	0.4 (0.2–0.8)		0.7 (0.3–1.2)	
	7	4.7 (3.6–5.9)	0.1 (0–0.5)		0.3 (0.1–0.7)	
	8	1 (0.5–1.7)	0 (0–0.3)		0.1 (0–0.4)	
	9	0.1 (0–0.6)	0 (0–0.3)		0 (0–0.3)	
40 %	5	40.1 (38–42.2)	0.7 (0.4–1.1)	0.06 (0.04–0.09)	1.2 (0.8–1.8)	0.11 (0.08–0.14)
	6	22.6 (20.6–24.7)	0.6 (0.3–1.1)		0.9 (0.5–1.5)	
	7	10.6 (9–12.5)	0.3 (0.1–0.7)		0.7 (0.3–1.2)	
	8	4 (3–5.3)	0.1 (0–0.4)		0.2 (0.1–0.7)	
	9	1.2 (0.6–2.1)	0 (0–0.4)		0.1 (0–0.5)	
	10	0.3 (0.1–0.9)	0 (0–0.4)		0 (0–0.4)	
	11	0.1 (0–0.6)	0 (0–0.4)		0 (0–0.4)	
50 %	5	42.1 (40–44.3)	0.7 (0.4–1.1)	0.08 (0.05–0.1)	1.3 (0.9–1.8)	0.14 (0.11–0.18)
	6	27.3 (25.1–29.5)	0.7 (0.4–1.2)		1.2 (0.7–1.8)	
	7	15.8 (13.9–17.9)	0.4 (0.2–0.9)		1 (0.5–1.7)	
	8	8.1 (6.6–9.8)	0.2 (0.1–0.6)		0.5 (0.2–1)	
	9	3.4 (2.4–4.8)	0.2 (0–0.6)		0.3 (0.1–0.9)	
	10	1.4 (0.7–2.5)	0.1 (0–0.5)		0.1 (0–0.7)	
	11	0.4 (0.1–1.1)	0 (0–0.4)		0 (0–0.5)	
	12	0.2 (0–0.8)	0 (0–0.5)		0 (0–0.5)	

Whilst the proportion of patients reclassified due to hs-cTnI assay imprecision was high for all CVs evaluated, the likelihood of those misclassified having a missed diagnosis of MI due to assay imprecision was very low – just 1 in 10,000 patients tested if the CV is 10 % at the single-sample rule-out threshold of <5 ng/L. Our findings were consistent for the secondary outcome of any myocardial injury on serial testing. A recent study across 35 hospital laboratories in Canada showed that less than one-third of the laboratories achieved a ≤10 % CV at the very low cTn cut-offs recommended in clinical pathways, suggesting reclassification due to imprecision is common in practice [5]. Our study provides complimentary insights into the clinical implications of assay imprecision, suggesting that even with substantial reclassification when applying a single-sample rule-out threshold, the probability of an adverse outcome in the High-STEACS trial as a consequence of misclassification is very low. Greater assay precision at these thresholds would reduce the likelihood of misclassification.
